# The Effect of Neoadjuvant Chemoradiotherapy on Whole-Body Physical Fitness and Skeletal Muscle Mitochondrial Oxidative Phosphorylation *In Vivo* in Locally Advanced Rectal Cancer Patients – An Observational Pilot Study

**DOI:** 10.1371/journal.pone.0111526

**Published:** 2014-12-05

**Authors:** Malcolm A. West, Lisa Loughney, Daniel Lythgoe, Christopher P. Barben, Valerie L. Adams, William E. Bimson, Michael P. W. Grocott, Sandy Jack, Graham J. Kemp

**Affiliations:** 1 Colorectal Surgery Research Group, Aintree University Hospitals NHS Foundation Trust, Liverpool, United Kingdom; 2 Department of Musculoskeletal Biology and MRC – Arthritis Research UK Centre for Integrated Research into Musculoskeletal Ageing (CIMA), Faculty of Health and Life Sciences, University of Liverpool, Liverpool, United Kingdom; 3 Magnetic Resonance and Image Analysis Research Centre (MARIARC), University of Liverpool, Liverpool, United Kingdom; 4 Critical Care Research Area, Southampton NIHR Respiratory Biomedical Research Unit, Southampton, United Kingdom; 5 Anaesthesia and Critical Care Research Unit, University Hospital Southampton NHS Foundation Trust, Southampton, United Kingdom; 6 Cancer Research UK Liverpool Cancer Trials Unit, University of Liverpool, Liverpool, United Kingdom; 7 Integrative Physiology and Critical Illness Group, Clinical and Experimental Sciences, Faculty of Medicine, University of Southampton, Southampton, United Kingdom; Pontificia Universidad Catolica de Chile, Chile

## Abstract

**Background:**

In the United Kingdom, patients with locally advanced rectal cancer routinely receive neoadjuvant chemoradiotherapy. However, the effects of this on physical fitness are unclear. This pilot study is aimed to investigate the effect of neoadjuvant chemoradiotherapy on objectively measured *in vivo* muscle mitochondrial function and whole-body physical fitness.

**Methods:**

We prospectively studied 12 patients with rectal cancer who completed standardized neoadjuvant chemoradiotherapy, recruited from a large tertiary cancer centre, between October 2012 and July 2013. All patients underwent a cardiopulmonary exercise test and a phosphorus magnetic resonance spectroscopy quadriceps muscle exercise-recovery study before and after neoadjuvant chemoradiotherapy. Data were analysed and reported blind to patient identity and clinical course. Primary variables of interest were the two physical fitness measures; oxygen uptake at estimated anaerobic threshold and oxygen uptake at Peak exercise (ml.kg^−1^.min^−1^), and the post-exercise phosphocreatine recovery rate constant (min^−1^), a measure of muscle mitochondrial capacity *in vivo*.

**Results:**

Median age was 67 years (IQR 64–75). Differences (95%CI) in all three primary variables were significantly negative post-NACRT: Oxygen uptake at estimated anaerobic threshold −2.4 ml.kg^−1^.min^−1^ (−3.8, −0.9), p = 0.004; Oxygen uptake at Peak −4.0 ml.kg^−1^.min^−1^ (−6.8, −1.1), p = 0.011; and post-exercise phosphocreatine recovery rate constant −0.34 min^−1^ (−0.51, −0.17), p<0.001.

**Conclusion:**

The significant decrease in both whole-body physical fitness and *in vivo* muscle mitochondrial function raises the possibility that muscle mitochondrial mechanisms, no doubt multifactorial, may be important in deterioration of physical fitness following neoadjuvant chemoradiotherapy. This may have implications for targeted interventions to improve physical fitness pre-surgery.

**Trial Registration:**

Clinicaltrials.gov registration NCT01859442

## Introduction

In the UK colorectal cancer is the third commonest cause of cancer death [1], [2]. In 2013, ∼9000 patients were diagnosed with rectal cancer (35% aged >75 y), of whom ∼4600 underwent major resection with a 90-day elective postoperative mortality of 2.5% [3]. 25% are locally advanced (Tumour, Node, Metastasis (TNM) stage - T3/T4N+) cancers (i.e. resection margin threatened) considered for neoadjuvant chemoradiotherapy (NACRT) to control local disease and to achieve tumour downsizing and negative resection margins [4]–[8]; however, external beam radiation and oral or intravenous fluoropyrimidines causes dose-limiting toxicity, reaching Grade 3–5 in 20% (Common Terminology Criteria for Adverse Events, Version 3.0). It is unknown to what extent NACRT affects physical fitness in this patient cohort.

Poor physical fitness, assessed by cardiopulmonary exercise testing (CPET), is linked to poor postoperative outcomes after major surgery [9]–[15]. CPET provides an integrated quantitative assessment of the cardiorespiratory system at rest and under the stress of maximal exercise, testing the physiological reserve required to withstand the stress of surgery. Subjective assessment tools have been used to predict surgical outcomes, but there is little evidence linking objectively-measured physical fitness and surgical outcome in this group. The UK National Bowel Cancer Audit found the American Society of Anaesthesiologists – Physical Status (ASA-PS) score (a categorical descriptor of fitness for surgery) to be the strongest predictor of death within 30 days of surgery [16]. Only two trials have suggested that rectal cancer patients with a higher subjective performance status (WHO Score >1) have worse post-operative outcome after combined chemotherapy or chemo-radiation and surgery [17], [18]. Studies investigating objective changes in physical fitness in patients receiving neoadjuvant cancer treatments are lacking [19]. We have previously demonstrated a significant reduction in objectively measured physical fitness with neoadjuvant chemotherapy in upper gastrointestinal cancer which was associated with reduced 1 year survival [20] and a similar reduction in fitness with neoadjuvant chemoradiotherapy in rectal cancer which was associated with in-hospital morbidity [21]. Whether and how this impaired physical fitness relates to changes in mitochondrial function is unknown.

Skeletal muscle mitochondrial function can be studied non-invasively *in vivo* using phosphorus magnetic resonance spectroscopy (^31^P MRS) [22]; this can usefully be combined with CPET measurements [23], [24] of whole-body fitness, to which muscle mitochondrial function makes a substantial contribution. Good correlations are observed between *in vivo and in vitro* measures of mitochondrial function in health and chronic conditions (e.g. type 2 diabetes) makes the assessment of mitochondrial function by ^31^P MRS an attractive and reliable modality, especially for repeated measurements [24]–[26].

The primary aim of this pilot study was to evaluate changes in objectively-measured physical fitness and skeletal muscle mitochondrial function after standardized NACRT, in patients scheduled for rectal cancer surgery. An exploratory aim was to observe changes in physical activity (PA) in the same patient cohort.

## Methods

### Patients and clinical methods

The protocol for this trial and supporting TREND checklist are available as supporting information; see Checklist S1 and Protocol S1. This nested mechanism pilot study forms part of a larger clinical trial which began in March 2011. Ethics approval for the main trial was given by the North West – Liverpool East Research and Ethics Committee (11/H1002/12) in March 2011, with a subsequent amendment (11/H1002/12c) adding ^31^P MRS measurements for this nested mechanism pilot sub-study approved in January 2012. The larger trial was registered with clinicaltrials.gov (NCT01325909 – March 2011), and initially this NCT registration was taken to cover all aspects of the larger trial, including the present pilot study. Subsequently the pilot study was registered separately (NCT 01859442 – May 2013), and as a result of this change of approach this specific registration post-dated the recruitment of the first patients reported here, for which studies commenced in October 2012. The authors confirm that all ongoing and related trials for this intervention are registered. Written informed consent was obtained from all patients. All patients were followed up until surgery, with the last patient exiting the study in October 2013.

We recruited consecutive patients between October 2012 and July 2013 who were referred to the Colorectal Multi-Disciplinary Team (MDT), age ≥18 y, with locally advanced resectable rectal cancer (circumferential resection margin threatened), scheduled for standardized NACRT on the basis of Tumour, Node, Metastasis (TNM) classification >T2/N+ with no distant metastasis [27] and WHO Performance Status <2 [28]. Predefined exclusion criteria were: inability to give informed consent, non-resectable disease, standard MR exclusion criteria, inability to exercise studies due to leg dysfunction, and patients who declined surgery or NACRT, or who received non-standard NACRT.

TNM staging involved flexible sigmoidoscopy for histological diagnosis, colonoscopy, chest, abdomen and pelvis computer-aided tomography (CT) and a 1.5T pelvic magnetic resonance imaging (MRI). All patients then underwent standardised NACRT for 5 weeks. Standardized radiotherapy consisted of 45 Gy in 25 fractions on weekdays using a 3-dimensional conformal technique with CT guidance. A boost dose was given (5.4 Gy in 3 fractions) to the primary tumour only. Oral capecitabine (825 mg.m^−2^) was given twice daily on radiotherapy days. No patients received brachytherapy. The colorectal multidisciplinary team (MDT) was blind to CPET results, which therefore did not influence perioperative management. All patients underwent total mesorectal excision (TME) surgery [29]. A defunctioning stoma was constructed at the discretion of the surgeon. No deviations from the protocol were encountered.

### CPET

CPET (Geratherm Respiratory GmbH; Love Medical Ltd, Manchester, United Kingdom) and the estimation of the estimated anaerobic threshold followed a standard protocol described in detail elsewhere [14]. Patient characteristics recorded included age, gender, height, weight, diagnosis, staging, surgical procedure planned, WHO classification, ASA-PS, and diagnoses of diabetes, ischaemic heart disease, cerebrovascular disease, or heart failure. Resting flow-volume loops were used to derive Forced Expiratory Volume over 1 second (FEV1) and Forced Vital Capacity (FVC). Ventilation and gas exchange variables included oxygen uptake (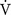
o_2_), ventilatory equivalents for oxygen and carbon dioxide (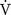
_E_/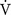
o_2_; 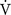
_E_/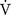
co_2_) and oxygen pulse (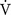
o_2_/heart rate), all measured both at estimated anaerobic threshold (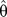
_L_) and at peak exercise.

NACRT associated toxicity and CPET-related adverse events were discussed at the weekly MDT meeting. Toxicity events were graded on the National Cancer Institute Common Terminology Criteria (version 3.0), and acute radiation-induced skin toxicity using the Radiation Therapy Oncology Group scoring system.

### Physical activity

PA was measured during a continuous 72 h period using a biaxial accelerometer (SenseWear armband BodyMedia Inc., Pittsburgh, USA), worn over the right triceps during weekdays at baseline and post-NACRT. Step count while active averaged over the whole 72 h was used as a measure of PA. All patients were instructed not to change their PA, and to continue performing normal activities of daily living throughout the study period.

### MRS methods

^31^P MRS assessments of muscle mitochondrial function in quadriceps were carried out using a Siemens 3T Trio MR scanner (Siemens AG, Erlangen, Germany) using an isometric knee extension exercise protocol. Subjects lay supine (secured with a Velcro strap across the hips) with the right knee flexed over a rigid foam support in a custom-built rig permitting isometric knee extension exercise against a strap across the anterior lower shin/ankle connected to an aluminium bar fitted with a strain gauge. ^31^P MRS data were acquired from right quadriceps muscle using a dual-tuned 18 cm/15 cm diameter ^31^P/^1^H surface coil (RAPID Biomedical, Rimpar, Germany), Velcro-strapped to the anterior thigh (midway between anterior superior iliac spine and patella). After automated set-up and manual shimming using tissue water signal, a 4-scan fully relaxed (TR = 10s) spectrum and a 32-scan partially saturated (TR = 2s) resting spectrum were collected. The exercise protocol consisted of 5 min rest followed by 2 bouts of isometric exercise (paced at 2 s on, 2 s off by an audible cue, exercise force being fed back visually via an LED display) each followed by 7 min recovery, while spectra were collected (TR = 2s) every 8 s. Two exercise intensities were used, corresponding to 70% and 90% of maximal voluntary contraction established in 3 brief trials prior to MRS acquisition (in pilot experiments these intensities gave acceptable PCr depletion with minimal acidification in typical subjects). Block MRS data output files from exercise-recovery acquisitions were converted to text using a specially-written MATLAB routine. All ^31^P MRS data were processed using the java-based Magnetic Resonance User Interface (jMRUI v.3.0), using the AMARES time-domain fitting algorithm. Data were fitted assuming Lorentzian lineshapes for phosphocreatine (PCr), inorganic phosphate (Pi) and ATP (β-ATP a 1∶2∶1 triplet, α-ATP and γ-ATP both 1∶1 doublets). The chemical shift of Pi relative to PCr was used by standard means to determine intracellular pH. PCr recovery time courses were fitted to a monoexponential function to estimate the recovery rate constant (k_PCr_ min^−1^): in the absence of appreciable changes in pH, this is an accepted measure of effective muscle mitochondrial function. As k_PCr_ did not differ significantly between the two exercise intensities, and as pH changes were small throughout, values of k_PCr_ are presented as mean of the two intensities [30].

All patients underwent CPET, PA monitoring and ^31^P MRS 2 weeks before NACRT (baseline) and immediately post-NACRT (post-NACRT) that is within 48 hours of finishing NACRT. CPET data were reported by two experienced assessors blind to patient demographics and data time-points. Any CPET or exercise-related adverse events were discussed at the weekly colorectal MDT meeting. ^31^P MRS analysis was performed blind to clinical details and CPET data.

Our primary outcome variables include 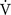
o_2_ at 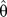
_L_, 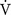
o_2_ Peak and k_PCr_, assessed with CPET and ^31^P MRS; an exploratory outcome variable is the number of steps whilst active, assessed by PA monitoring. A comparison of these variables is made between baseline and post-NACRT.

### Statistical methods

Our aim was to recruit 12 patients to a pilot study who would undergo standardised NACRT, CPET and ^31^P MRS scans at baseline (pre-NACRT) and immediately post-NACRT. No formal sample size calculation was performed as this study was designed as a nested pilot study: the target number was based on experience and reports of similar clinical ^31^P MRS studies and pragmatic considerations of recruitment logistics and funding. Descriptive statistics are reported as mean (SD) or median and inter-quartile range (IQR) depending on the distribution and categorical statistics as frequency (percentage). Normality was assessed by the Shapiro-Wilk test. Pre- and post-NACRT data were compared using paired t-tests.

For the primary analysis, baseline and post-NACRT measurements for 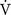
o_2_ at 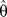
_L_, 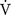
o_2_ Peak and k_PCr_ were compared using a paired t-test as an intention to treat. Formal comparisons were considered to be statistically significant at p<0.05. Spearman's correlation coefficient (r) was used to describe the strength of association between changes in 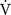
o_2_ at 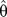
_L_ and change in haemoglobin. For PA these comparisons were considered as exploratory and tested against the uncorrected 5% significance level; the need to square-root transform PA makes it impossible to recover the differences and confidence intervals on a meaningful scale, so only p-values and predicted means are presented. These analyses were conducted using Stata version 12 (StataCorp. 2011. *Stata Statistical Software: Release 12*. College Station, TX: StataCorp LP.)

## Results

### Patient flow and characteristics

**Figure 1:**
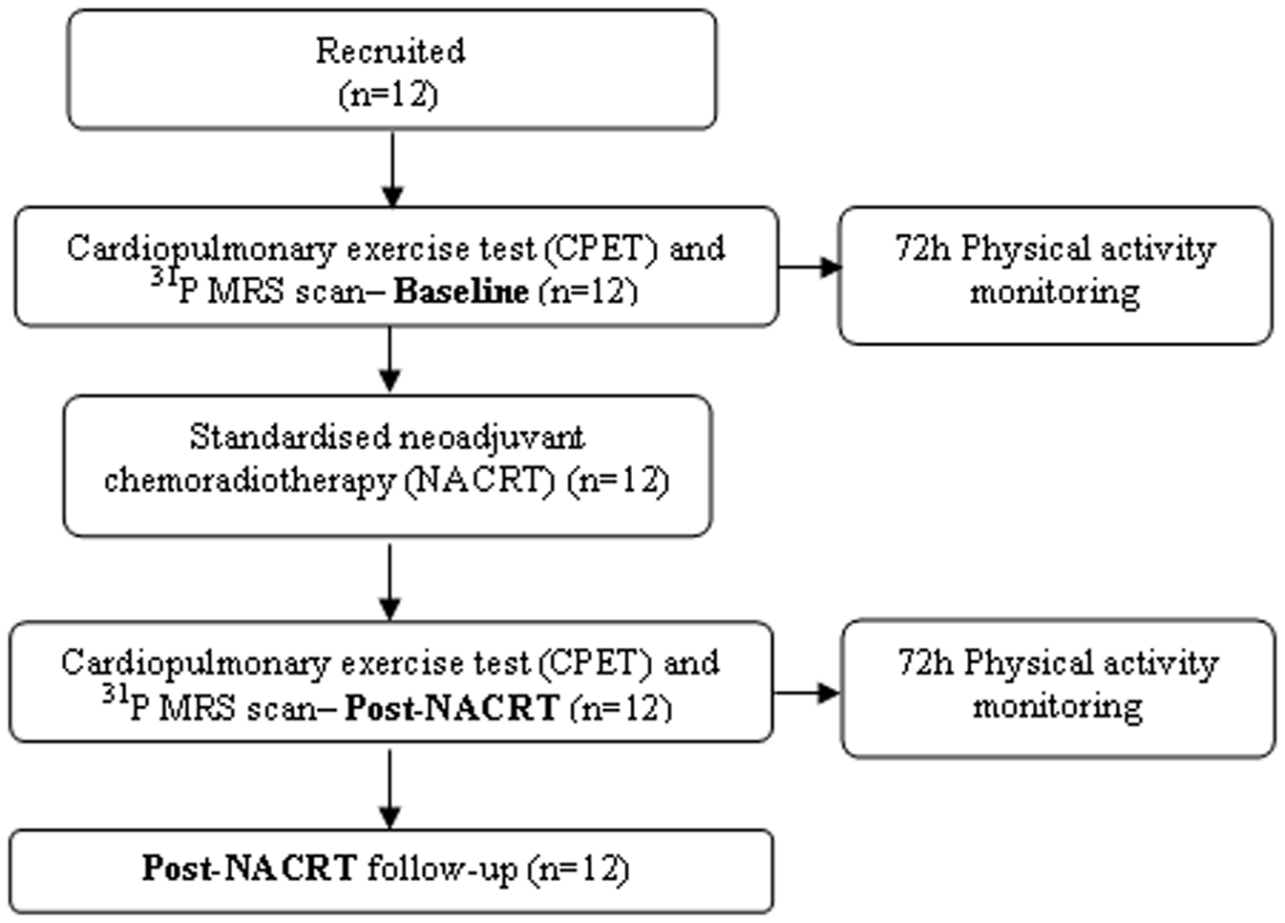
Showing flow of patients through the study protocol. Post-NACRT tests carried out within 48±5 hours of finishing NACRT period.

Fifteen patients were eligible for surgery, of which 3 were recruited into a different trial; 12 patients (10 males and 2 females) were recruited and underwent CPET and ^31^P MRS prior to starting a standardised course of NACRT (Figure 1). All CPETs were carried out 48±5 hours before the ^31^P MRS scan both at baseline and post-NACRT. All patients underwent CPET and ^31^P MRS immediately after (post-NACRT) finishing NACRT (at a maximum of 48±5 hours). Table 1 describes baseline patient characteristics and Table 2 describes patient characteristics between baseline and post-NACRT. There were no significant changes in BMI or lung function following NACRT, but there was a small but significant fall in haemoglobin. 83% of diagnosed rectal cancers were T3 with threatened circumferential resection margins.

**Table 1:** Baseline patient characteristics.

	Mean (SD)
Age (years)	69 (10)
	**Number (%)**
Gender Male:Female	10 (83): 2 (17)
Currently smoking	2 (17)
Past medical history	
Diabetes	1 (8)
Ischaemic heart disease	2 (17)
Heart failure & cerebrovascular disease	0
American Society of Anaesthesiologists (ASA) score	
1	6 (50)
2	6 (50)
World Health Organisation performance status	
0	9 (75)
1	3 (25)
Tumour distance from anal verge (cm)	
<5.0 cm	6 (50)
5.1–10.0 cm	5 (42)
>10.1 cm	1 (8)
International Union against Cancer Tumour Node Metastasis (TNM) MRI staging	
cT3	10 (83)
cT4	2 (17)
cN0	3 (33)
cN1	4 (17)
cN2	5 (50)
cM0	12 (100)

**Table 2:** Patient characteristics at Baseline and post-NACRT.

	Baseline	Post-NACRT	Mean difference	P
	mean (SD)	mean (SD)	(95% CI)	
BMI (kg.m^−2^)	26.8 (3.9)	26.3 (3.2)	−0.5 (−0.5, 1.6)	0.291
FEV1 (l)	3.0 (0.7)	2.9 (0.8)	−0.1 (−0.1, 0.4)	0.153
FVC (l)	4.3 (0.8)	4.2 (0.9)	−0.2 (−0.1, 0.4)	0.188
FEV1/FVC (%)	72 (7.3)	73 (7.3)	1.2 (−3.1, 0.8)	0.215
Haemoglobin (g.dl^−1^)	13.2 (1.5)	12.9 (1.5)	−0.4 (0.5, 0.7)	**0.034**

### Chemoradiotherapy and acute toxicity

The mean cumulative dose of capecitabine was 96% (range 84–100%) of the planned treatment dose; 1 patient needed dose reduction. All but 1 patient received at least 45 Gy radiotherapy, and all completed the full 25 fractions. 2 patients (including 1 receiving a diverting stoma because of obstructive symptoms prior to NACRT) experienced grade 3 toxicity, notably diarrhoea and radiation dermatitis, but no grade 4 toxicity was reported.

### The effect of NACRT on physical fitness, physical activity and mitochondrial function

**Figure 2:**
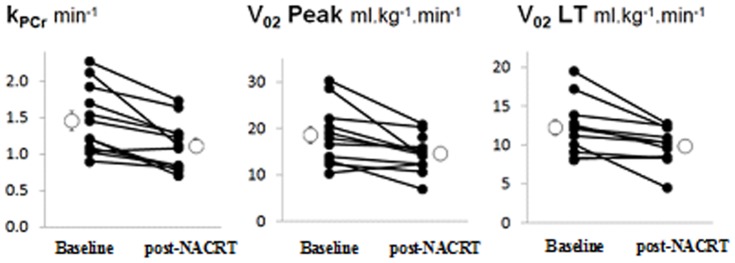
^31^P MRS (k_PCr_) and CPET (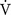
o_2_ at 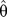
_L_ and 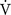
o_2_ at Peak) data at Baseline (before NACRT)) and at 48±5 hours post-NACRT: lines link data-points (closed circles) for individual patients, and open circles show overall mean±SEM. Mean changes (SEM) between baseline and post-NACRT are for k_PCr_ −0.4(0.1) min^−1^, p = 0.001; for 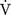
o_2_ at 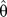
_L_ −2.4(0.7) ml.kg^−1^.min^−1^, p = 0.004; and for 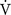
o_2_ Peak −4.0(1.3) ml.kg^−1^.min^−1^, p = 0.011.

**Figure 3:**
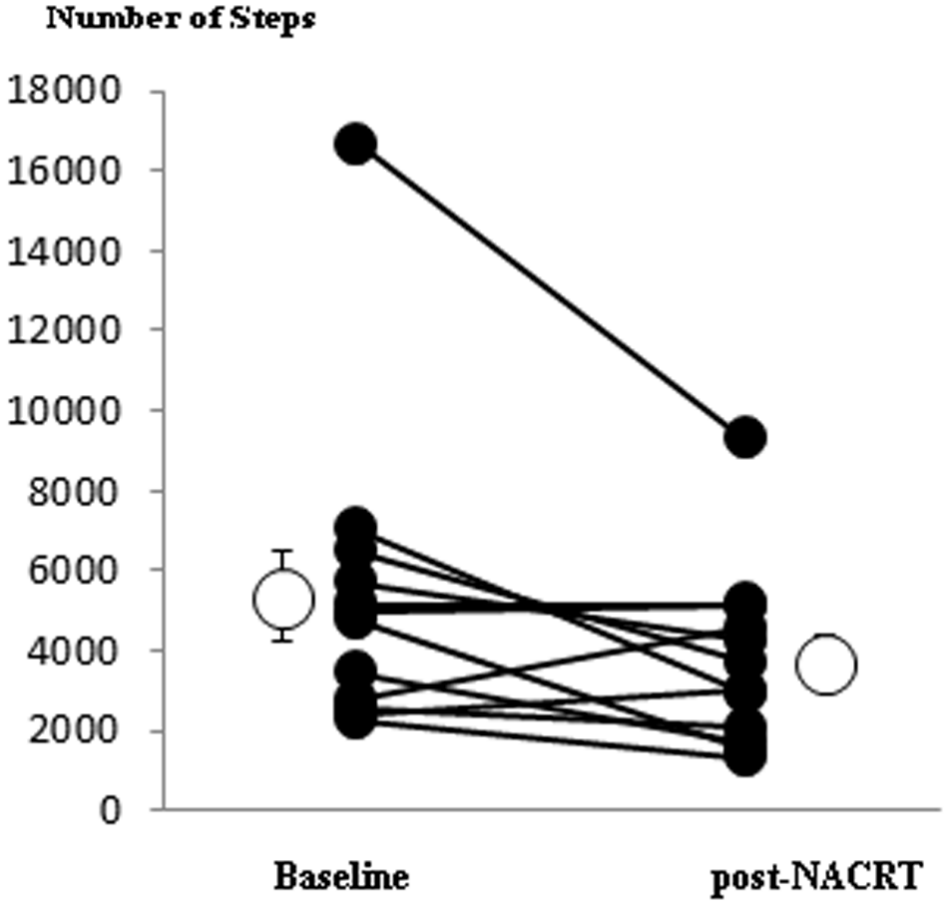
Averaged number of steps at Baseline (before NACRT)) and at 48±5 hours post-NACRT: lines link data-points (closed circles) for individual patients, and open circles show overall mean±SEM. Mean change (SEM) between baseline and post-NACRT is −1627(712) steps, p = 0.039.

Table 3 shows CPET, PA and ^31^P MRS-derived variables pre- and post-NACRT. No exercise or MRS adverse events were encountered until end of follow-up. Post-NACRT, there were significant decreases in both absolute (ml.min^−1^) and relative (ml.kg^−1^.min^−1^) 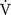
o_2_, in O_2_ pulse at 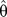
_L_ and at Peak exercise, in baseline heart rate, in k_PCr_ and in the number of steps (although this is strongly influenced by an apparent outlier; difference in the number of steps was not significant after the outlier was removed); Figure 2 shows mean and individual values of k_PCr_, 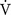
o_2_ at 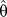
_L_ and 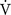
o_2_ at Peak, while Figure 3 shows the mean number of daily steps. 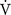
_E_/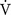
co_2_ and work rates at 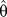
_L_ and Peak exercise did not change. No significant relationship was found between the change in 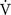
o_2_ at 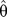
_L_ and change in haemoglobin (r = 0.27; p = 0.396).

**Table 3:** Patient data at Baseline and post-NACRT.

	Baseline	Post-NACRT	Mean difference	P
	mean (SD)	mean (SD)	(95% CI)	
k_PCr_^1^ (min^−1^)	1.5 (0.5)	1.1 (0.3)	−0.4 (−0.5, −0.2)	**0.001**
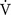 o_2_ at 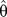 _L_ (ml.kg^−1^.min^−1^)	12.3 (3.4)	9.9 (2.2)	−2.4 (−3.8, −0.9)	**0.004**
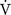 o_2_ at 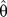 _L_ (L.min^−1^)	0.9 (0.3)	0.7 (0.2)	−0.2 (−0.1, −0.3)	**0.003**
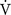 o_2_ Peak (ml.kg^−1^.min^−1^)	18.7 (6.1)	14.7 (4.0)	−4.0 (−6.8, −1.1)	**0.011**
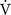 o_2_ Peak (L.min^−1^)	1.4 (0.5)	1.1 (0.3)	−0.3 (−0.1, −0.5)	**0.013**
O_2_ pulse at 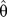 _L_ (ml.beat^−1^)	8.9 (2.9)	7.7 (2.2)	−1.2 (−0.2, −2.1)	**0.022**
O_2_ pulse at Peak (ml.beat^−1^)	10.5 (3.6)	8.7 (2.2)	−1.8 (−0.3, −3.3)	**0.024**
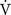 _E_/ 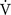 co_2_ at 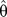 _L_	34.5 (6.4)	34.2 (4.3)	−0.3 (2.5, −3.1)	0.825
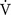 _E_/ 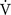 co_2_ at Peak	35.9 (4.6)	37.1 (4.8)	0.3 (−3.7, 1.4)	0.080
Average number of steps	5352 (3913)	3725 (2217)	−1627 (−59, 3195)	**0.039**

Abbreviations: k_PCr_, post-exercise phosphocreatine recovery rate constant; 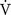
o_2_ at 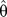
_L_, oxygen uptake at estimated anaerobic threshold; 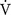
o_2_ Peak, oxygen uptake at peak exercise; O_2_ pulse at 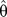
_L_, oxygen pulse at estimated anaerobic threshold; O_2_ pulse at Peak, oxygen pulse at peak exercise; 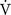
_E_/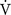
co_2_ at 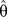
_L_, ventilatory equivalents for carbon dioxide at estimated anaerobic threshold; 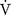
_E_/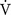
co_2_ at 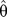
_L_, ventilatory equivalents for carbon dioxide at peak exercise; Work rate at 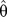
_L_, work rate at estimated anaerobic threshold; Work rate at Peak, work rate at peak exercise.

## Discussion

This is the first study to identify mitochondrial abnormalities accompanying changes in physical fitness following neoadjuvant chemoradiotherapy. The benefits of NACRT for locally advanced rectal cancer are improved local disease control and possibly overall and cancer-specific survival; however its effect on objectively measured physical fitness has not been measured, nor has the mechanism of this been explored *in vivo*.

This pilot study shows a significant reduction in both whole-body physical fitness measured by CPET (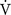
o_2_ at 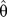
_L_ −2.36 ml.kg^−1^.min^−1^, 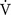
o_2_ at Peak −3.95 ml.kg^−1^.min^−1^) and *in vivo* muscle mitochondrial function k_PCr_ (−0.34 min^−1^) between baseline and post-NACRT. We also found a significant decline in PA with NACRT (−1627 steps). This acute decline in mitochondrial function may account for the rapid loss in fitness and activity over the neoadjuvant treatment period, clinically important in the context of fitness for surgery and perioperative risk however causality cannot be established as controlling for PA in a clinical setting is difficult, and statistical adjustment for PA in a small patient group is not feasible. These findings however illuminate a potential mechanistic link that might be contributing to the changes in objectively measured whole body physical fitness with NACRT, consistent with the results of our earlier pilot study [21].

Cancer-induced cachexia can cause major loss of skeletal muscle, resulting in fatigue and higher mortality [31],[32]. In our cohort cancer progression is not a contributing factor as tumours were downstaged. Furthermore BMI and weight remained stable. The small but statistically significant fall in haemoglobin is unlikely to be functionally relevant, and showed no correlation with the CPET or ^31^P MRS changes.

Several publications postulate mechanisms by which chemotherapy may contribute to skeletal muscle dysfunction [33]. Oxidative damage [34] resulting from doxorubicin-based chemotherapy in haematological malignancies causes sarcopaenia [35], up-regulation of E3 ubiquitin-ligase/MAFbx [35] and mitochondrial death [36]. Drugs with a quinone moiety can directly interact with oxygen to generate reactive oxygen species (ROS) causing oxidative stress-mediated injury to cardiac muscle, kidneys and brain tissue [37], while other chemotherapeutic agents decrease antioxidant levels [37]; however these chemotherapy regimens were not used in this cohort. At subcellular levels, mitochondria are major targets for chemotherapy-induced oxidative stress [36]. Chemotherapy is known to affect cardiorespiratory (causing exercise intolerance) [38] and microcirculatory function [39], PA [33], but these multifactorial physiological mechanisms remain elusive.

Mitochondrial function measured by ^31^P MRS is impaired in a variety of chronic diseases, as well as primary mitochondrial disease. In peripheral arterial occlusive disease [30], [40] this mainly reflects impaired O_2_ delivery to the muscle. In cardiac failure [41] and COPD [42], [43] it is likely to reflect a multifactorial pathology including reduced PA, mitochondrial density [44] and ROS mechanisms. Similar mechanisms might mediate the decline in physical fitness in our cohort. The acute decline in PA seen might also contribute to changes in mitochondrial function and physical fitness. A better understanding and quantification of the potential mechanisms involved if oxidative injury is present in this patient cohort is essential to design intervention strategies that will attenuate the toxicity of chemoradiotherapy agents without compromising their anticancer effects [36]

Our findings have potential clinical implications because reduced physical fitness is associated with increased perioperative morbidity and mortality after major intra-abdominal surgery, especially colorectal surgery [14], [15], [45] Our data provides the first direct evidence that the benefits of NACRT in tumour downsizing may be at least partly offset by increased perioperative risk as a result of reduced physical fitness, related to reduced skeletal muscle mitochondrial function and activity however causality cannot be established. This proposed mechanism merits further investigation, as does the possibility of preventive interventions e.g. by exercise training during the pre-operative period (currently the focus of our published exercise training study [46]). This is an interventional pilot study in the same patient cohort scheduled to undergo standardised NACRT and a 6-week structured, tailored exercise training programme (exercise group n = 22) or a control period (n = 13). Here 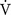
o_2_ at 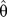
_L_ significantly reduced between baseline and post-NACRT (−1.9 ml.kg^−1^.min^−1^). In the exercise group 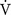
o_2_ at 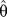
_L_ significantly improved between post-NACRT and week 6 post-NACRT (+2.1 ml.kg^−1^.min^−1^) whereas control group values were unchanged (−0.7 ml.kg^−1^.min^−1^). Furthermore a randomised controlled study investigating the potential benefits in physical fitness and quality of life of a 9-week structured responsive endurance training programme following NACRT prior to elective rectal cancer surgery (PB-PG-0711-25093) is currently recruiting.

This study demonstrates an acute decline in objectively-measured physical fitness, activity as well as a decline in mitochondrial function using validated and robust methodology. Particular strengths of our study are the low risk of confounding by indication [47], the blinded physiological evaluations, the standardization of the NACRT and the homogenous cancer cohort. Limitations lie in the observational design, the small sample size of what was designed as a pilot study and that no adjusting for multiple testing was performed.

In conclusion, NACRT before major rectal cancer surgery significantly reduces physical fitness objectively assessed by CPET and muscle mitochondrial function assessed by ^31^P MRS. The existence of abnormalities at both skeletal-muscle and whole-body level may perhaps suggest a potential mechanistic relationship in NACRT which merits further investigation, which could potentially inform development of tailored interventions in the perioperative period to improve both physical fitness and mitochondrial function in patients with operable rectal cancer.

## Supporting Information

Checklist S1 **TREND statement checklist.**
(PDF)

Protocol S1 **Study protocol for patients consented to this trial.**
(DOCX)
