# Supplementary material for: The Effect of Neoadjuvant Chemoradiotherapy on Whole-Body Physical Fitness and Skeletal Muscle Mitochondrial Oxidative Phosphorylation In Vivo in Locally Advanced Rectal Cancer Patients – An Observational Pilot Study
Source: PLoS One. 2014 Dec 5;9(12):e111526. doi: 10.1371/journal.pone.0111526 (PMC4257525; doi:10.1371/journal.pone.0111526)
Supplement: Protocol S1 — Study protocol for patients consented to this trial. (DOCX) [file pone.0111526.s002.docx]

## Aintree University Hospitals


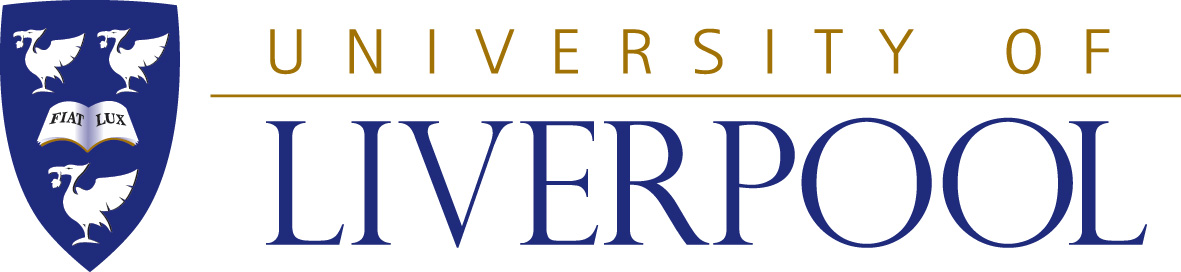

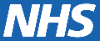


NHS Foundation Trust

**University Hospital Aintree**

Lower Lane

Study Number:

Patient Information Number:

Liverpool

L9 7AL

Tel: 0151-525-5980

Does short-term exercise intervention improve pre-operative physical fitness following neoadjuvant chemoradiotherapy in colorectal cancer patients?

Study Protocol For Patients Consented For Mechanism Pilot Study

**Overview**

The present study is a randomised control trial investigating the effects of a short six week exercise training program in colorectal cancer patients following neoadjuvant chemoradiotherapy prior to elective colorectal cancer surgery. All patients will attend 5 sets of appointments at the hospital for diagnostic tests and **3 appointments at the University of Liverpool Magnetic Resonance and Image Analysis Research Centre (MARIARC)** (listed below). **Three** appointments will be prior to chemoradiotherapy treatment, and the other **4** will be after chemoradiotherapy. Two of these 5 appointments are part of routine care. The first **4** appointments will last about an hour. Patients will complete 3 quality of life questionnaires on recruitment, during chemotherapy, before and 4-6 weeks after surgery. These 3 questionnaires will take 20 minutes to fill in. The fourth and fifth appointments will consist of Computed Tomogram (CT) and Magnetic Resonance Imaging (MRI) scans in our radiology department to assess cancer staging. These appointments will last about 1 hour 30 minutes.

Patients will be randomised (3:1 ratio) to an intervention and a control group**.** The patients randomized to the intervention group will attend a total of 18 tailored exercise sessions (Three 40 minute sessions for six weeks) after their 6 weeks of chemoradiotherapy treatment. Where possible the exercise training sessions will be arranged to fit in with other appointments at the hospital. Patients randomized to the control group will be given best exercise advice and will be unsupervised. They will only attend an extra 3 sessions were a cardiopulmonary exercise test and VO2 Kinetics test will be performed. Patients will also be invited to attend 3 health related quality of life interviews at week 0, 3 and 6 during their exercise programme. These appointments will be directly before or after their exercise sessions to minimise hospital attendences.

Following surgery only routine clinically relevant observational data will be collected. These data will relate to hospital length of stay, the level of care required following surgery, post-operative morbidity survey (POMS) and the recovery process. Most of this information can be accessed from patient notes and on the electronic patient records system.

We aim to recruit 52 patients who will undergo long-course chemoradiotherapy and elective colorectal cancer surgery as an intention to treat. This sample size is calculated by using a 2 sample t-test, with 80% power to detect an estimated increase of 10% in VO2, based on the study of Kothman et al. Following Kothman et al we also estimated a drop-out rate of about 20%. **A subgroup of these patients (6 control and 6 exercise intervention patients) will be ask to consent separately for the muscle biopsies, the ^31^Phosphorus Magnetic Resonance Spectroscopy (^31^P MRS) scans and the blood samples.**

**HYPOTHESIS**

1. Interval exercise training will maintain or improve fitness (measured by anaerobic threshold) in patients undergoing neoadjuvant chemoradiotherapy.

2. Interval exercise training is safe and feasible in patients undergoing neoadjuvant chemoradiotherapy awaiting colorectal cancer resection.

3. Interval exercise training will improve other measures of physical fitness measured in the CPET and the oxygen uptake kinetics test.

4. Interval exercise training will improve quality of life in patients undergoing neoadjuvant chemoradiotherapy.

5. Improvements in physical fitness will reduce postoperative complications following major colorectal cancer surgery.

6. Does physical activity, measured by Sensewear Pro 3 activity monitors, decrease during neoadjuvant chemoradiotherapy and can this decrease be attenuated by an exercise training program?

7. Can we find an optimal time for surgery when fitness and cancer downstaging are at their best?

**HYPOTHESIS for Mechanism Pilot Study**

1. **To explore the exponential rate constant of post-exercise phosphocreatine recovery.**
2. **To explore the alteration in cellular and mitochondrial energetics eg. Change in mitochondrial numbers, change in mitochondrial activity and respiration.**

**Appointments**

**Cardiopulmonary Exercise Test (CPET)**

Six symptom limited Cardiopulmonary Exercise Tests (CPET) will be performed as part of the study occurring before and after the chemoradiotherapy treatment. Specifically, this involves cycling on an exercise bike for 8-12 minutes. Starting with a very low resistance on the pedals the patient will pedal at 60 revolutions per minute. After 3 minutes of cycling, the resistance will gradually increase at a constant rate until the patient can no longer turn the pedals at the required speed. The patient’s heart will be monitored by an electrocardiogram (ECG). The patient will wear a soft rubber mask in order to continuously sample expired air with an online breath by breath gas analyser. This test is common practice prior to operations and provides an accurate measure of physical capacity. A Near Infrared Spectroscopy (NIRS) probe will be attached to the thigh to non-invasively measure oxygen utilisation in the muscle by shining a light on to the thigh. Each CPET appointment will last approximately one hour. BORG scores will be assessed at intervals.

**Pulmonary Function Tests (PFT’s)**

The patients will perform pulmonary function tests (breathing tests). The breathing tests will be performed before and after the chemoradiotherapy treatment.

**VO2 kinetics test**

Patients will complete 2 submaximal / moderate exercise bouts on a cycle ergometer with 15 minutes rest between each bout. The test will consist of cycling at 10W for 3 minutes before the workload is abruptly increased to a workload equivalent to 80% of the estimated lactate threshold (estimated from the results of CPET) for a further 6 minutes. A NIRS probe will be attached to the thigh to non-invasively measure oxygen utilisation in the muscle (same as CPET). One of these tests will be done 72 hours after the first CPET test, whilst the other one will be incorporated into the exercise protocol (exercise session 17).

**Activity Monitoring**

Activity levels will be monitored by using a SenseWear Pro 3 activity monitor. Patients will wear an armband on the upper part of their dominant arm for a 3 day period. This will occur before, during chemoradiotherapy, during exercise training and 9 weeks post-exercise training. This will be done to assess changes in activity during the research process. These monitors will be fitted on routine care appointments.

**Muscle Biopsy**

**Muscle biopsies will be extracted using forceps, in each case approximately 20mg will be extracted. Muscle biopsies will be using an aseptic technique and local anaesthetic (1% lignocaine). Although there will be some stiffness following the biopsy (similar to a "dead leg"), this will disappear after 12- 14 days. These samples will take place prior to neoadjuvant chemoradiotherapy (NACRT), immediately after NACRT (at Week 0 prior to starting the exercise program), and on completion of the exercise programme (Week 6) as detailed in the flow-chart below.**

**Following extraction, muscle samples will be analysed for the following factors:**

- **Electron transport chain complexes**
- **Citrate synthase**
- **Mitochondrial function and respiration**

**These would give us an initial idea of whether mitochondria are being lost, or alternatively whether there is targeted complexes 1 and 3. We will also be looking for changes in:**

- **UCP3 uncoupling protein, thought to play a role in antioxidant defense or defense against lipotoxicity, but uncouples oxidative phosphorylation decreasing efficiency.**
- **PGC1alpha transcriptional regulator driving mitochondrial biogenesis**
- **PPARalpha fatty acid activated transcription factor drives expression of fatty acid oxidation enzymes (and UCP3)**
- **NRF1 nuclear respiratory factor 1, transcription factor driving expression of respiratory enzymes in mitochondria**
- **Mitofusin 2 drives mitochondrial fusion and is essential for maintaining mitochondrial morphology**
- **HIF1alpha transcription factor induced by hypoxia/oxidative stress.**

**Phosphorus magnetic resonance spectroscopy (^31^P MRS)**

**^31^P MRS studies of skeletal muscle bionergetics will be carried out on the 3T Siemens Trio scanner at the University of Liverpool Magnetic Resonance and Image Analysis Research Centre (MARIARC). This scan will take place prior to neoadjuvant chemoradiotherapy (NACRT), immediately after NACRT (at Week 0 prior to starting the exercise program), and on completion of the exercise programme (Week 6), as detailed in the flow-chart below.**

**Completion of an MR safety screening questionnaire will be performed on consent to the study to determine eligibility. On arrival at MARIARC the subjects will change into a hospital gown and proceed to the scanning room. The subject will lie flat on the scanner bed with the right leg semi-flexed over a custom-built exercise rig, with the ankle restrained by a velcro strap, and a ^31^P MR surface coil strapped to the anterior part of the thigh. A localising MR image will be acquired to assess coil placement. ^31^P MR scanning will be performed at rest, during and after short bouts of isometric knee-extension exercise at a rate of 0.5 Hz at various intensities: normally 2-5 min at each of 60% and 90% of maximum voluntary contraction, although this may be adjusted in preliminary pilot studies in this new patient group). Studies will take no more than 1 hour in the MR scanner.**

**^31^P MRS data will be analysed to quantify muscle cell pH, phosphocreatine (PCr) and inorganic phosphate (Pi), and kinetic analysis performed of the time-resolved data. The primary endpoint is the exponential rate constant of post-exercise PCr recovery, a measure of muscle mitochondrial function.**

**Standard MR exclusion criteria will apply (cardiac pacemaker, metal in eye, vascular clips, actual or possible pregnancy).**

**Study consent forms and MR screening forms will be kept in a locked filing cabinet. Names of subjects will be recorded, as is standard, in the MARIARC scanner logbook. Data from the studies will be kept securely on password-protected computers under coded identifiers only, without personal identifiers.**

**The main risks to the subjects are those related to MR. The use of a high magnetic field can cause injury (i.e. if the body contains any implanted magnetic material). Standard MR exclusion criteria will apply, and standard MR safety procedures will be strictly adhered to. All sequences are within NRPB guidelines for radiofrequency power deposition. MR scans are noisy, so subjects wear ear plugs. Safety is continually monitored both by the researchers and independently by responsible MARIARC personnel.**

**During scanning the subject will be continually monitored from the control room, and can abort at any time using a hand-held alarm. There will always be first aid- and resuscitation-trained personnel in the vicinity.**

**All relevant training and induction will be carried out. Guidelines and safety protocols have been made available to all investigators. The study will be managed, documented, monitored and reported according to standard MARIARC procedures. All aspects of the study will be monitored by the PI.**

**Blood tests**

**10mls of blood will be taken from each patient prior to neoadjuvant chemoradiotherapy (NACRT), immediately after NACRT (at Week 0 prior to starting the exercise program), and on completion of the exercise programme (Week 6). This will be processed as per Annex 1.**

**EXERCISE PROTOCOL – Intervention group**

Patients assigned to the intervention arm of the study will participate in a 6 week in-hospital, supervised exercise training program on a cycle ergometer. The training program will involve short periods of exercise at a high intensity interspersed with short periods of exercise at a moderate intensity. This mode of exercise training is termed aerobic interval training.

Exercise intensities during the interval exercise training program are specific to each patient and will be derived from the CPET test. Moderate intensity exercise is recognised as exercise below the anaerobic threshold. Patients will exercise at power outputs calculated to require 80% of the VO2 achieved at the anaerobic threshold (80%AT) for moderate intensity exercise. Heavy exercise is recognised as 50% of the difference between the VO2 at the anerobic threshold and the VO2 at peak oxygen consumption (50%∆). Patients will exercise at power outputs calculated to require 50%∆ for high intensity exercise.

Patients will attend 3 exercise sessions per week for 6 weeks. Each session will last <1 hour. Patients will be screened prior to each session to ensure that it is safe to perform exercise. Exercise training will involve repeated exercise bouts of 3 minutes at a moderate exercise intensity

(80%AT) followed by 2 minutes of exercise at a high intensity (50%∆) for a predetermined amount of time. In the first week of training, patients will perform the interval exercise protocol repeatedly

for 20 minutes (i.e. 4 repeated bouts of 3 minutes 80%AT and 2 minutes 50%∆). After the first week, the length of the training session will increase to 30 minutes for the remaining 5 weeks. 5 minutes of unloaded cycling will be performed at the start and end of the protocol for a warm-up and cool down. Blood pressure will be monitored every 3 minutes throughout exercise. Exercise sessions can be terminated by the patient at any time, and will be terminated by the supervisor of the test if any of the criteria to stop the test are met, based on the ATS CPET safety guidelines.

Patients will perform a CPET on the 1^st^, 7^th^ (session 1 of week 3) and 18^th^ exercise sessions (session 2 of week 6). Patients will be allowed 15 minutes to recover from the CPET and then asked to perform 10 minutes of the interval training (i.e. 2 repeated bouts of 3 minutes 80%VT and 2 minutes 50%∆). Exercise intensities for training will be adjusted based on the CPET data from the 7^th^ exercise session. Results from the CPET on the 18^th^ exercise session will be used to determine changes in exercise performance.

**Control group**

Patients assigned to the control arm of the study will be given best exercise advice. These will be unsupervised and out of hospital. They will also have 3 CPET tests; at week 0, one in week 3 and one in week 6 to coincide with the CPET tests of the activity group. A VO2 Kinetics test will be done also on week 6, 72 hours after the CPET test.

**Quality of Life Questionnaires**

Patents will be asked to fill in 3 health related quality of life questionnaires (SF-36, EQ-5D and EORTC QLQc30-v3). These will take no longer than 20 minutes to fill in. They will be given or posted to the patients to minimize inconvenience. They will coincide with patient recruitment, 3 weeks into chemoradiotherapy, 9-weeks after chemoradiotherapy and 4-6 weeks after surgery.

**Quality of Life Interviews**

During their 6 weeks post-chemoradiotherapy, patients will be asked to attend 3 quality of life interviews. This is irrespective of randomization. Patients will be interviewed by a dedicated sports and exercise psychologist who is part of the research team. Interviews will be recorded and will take place in a private clinic room. For the exercise intervention patient, interviews will be before or after an exercise session. For the control group, interviews will be before the CPET tests at week 0, 3 and 6 post-chemoradiotherapy. All interviews will last no more than 1 hour. Questions asked at the interviews are attached at the bottom of the protocol.

**Radiological Appointments**

After the exercise protocol all patients will undergo an abdominal and pelvic CT and MRI scan at 9 weeks after their chemoradiotherapy for restaging purposes. On the same appointment the patients will have a CPET test to assess their level of fitness. This is all part of the standard care pathway. At 14 weeks after chemoradiotherapy all patients will have a restaging MRI scan of their abdomen and pelvis to assess any further cancer down staging. On this occasion they will also undergo their final CPET test.

**Following Surgery**

Following surgery, information will be collected with regards to the length of stay in the hospital, the level of care that the patient received during this time and the recovery process. A Post-Operative Morbidity Score (POMS) will be recorded at 3, 5, 7 and 14 days after surgery. Resource utilization (e.g. hospital bed utilization) will also be used to measure post-operative outcome. All information collected following surgery will be carried out by observation only.

The complete sequence of tests

will be as follows:

Potential patient identified by the surgical team

Post-diagnosis out-patient visit + patient information leaflet discussed

Activity Monitoring

(3 days) and QOL questionnaires

Written consent taken

CPET and Pulmonary Function Tests

(1 – 2 weeks following diagnosis)

**STANDARD CARE PATHWAY**

**31 p MRS scan**

**Muscle Biopsy and Blood tests**

Neo-adjuvant chemoradiotherapy

(Normally lasting a total of 5-6 weeks)

**STANDARD CARE PATHWAY**

Activity Monitoring

(3 days) and QOL questionnaires

**Muscle Biopsy and 31 p MRS scan**

**Exercise Intervention Group**

(CPET at 0, 3 and 6 weeks)

Supervised – in hospital

QOL interview at 0, 3 and 6 weeks

**Muscle Biopsy and 31 p MRS scan at week 6**

**Control Group**

(CPET at 0, 3 and 6 weeks)

Unsupervised – out of hospital

QOL interview at 0, 3 and 6 weeks

**Muscle Biopsy and 31 p MRS scan at week 6**

Activity Monitoring

(3 days)

oxygen

Activity Monitoring

(3 days) and QOL questionnaires

PFT’s, CPET and Abdominal and Pelvic CT and MRI scans

(9 weeks post-chemoradiotherapy)

**STANDARD CARE PATHWAY**

CPET and Pelvic MRI scan

(14 weeks post-chemotherapy)

Surgery

QOL questionnaires (4-6 weeks)

**Exercise Intervention Protocol**

**Week 1 (Interval exercise training sessions 1-3)**

| **20 minutes exercise** | | | | | | | |
| --- | --- | --- | --- | --- | --- | --- | --- |
| **3 mins 80%VT** | **2 mins 50%∆** | **3 mins 80%VT** | **2 mins 50%∆** | **3 mins 80%VT** | **2 mins 50%∆** | **3 mins 80%VT** | **2 mins 50%∆** |

**Weeks 2 – 6 (Interval exercise training sessions 4-6 and 8-16)**

| **30 minutes exercise** | | | | | | | | | |
| --- | --- | --- | --- | --- | --- | --- | --- | --- | --- |
| **3 mins 80%VT** | **2 mins 50%∆** | **3 mins 80%VT** | **2 mins 50%∆** | **3 mins 80%VT** | **2 mins 50%∆** | **3 mins 80%VT** | **2 mins 50%∆** | **3 mins 80%VT** | **2 mins 50%∆** |

**VO2 kinetics test – Session 17**

**Interval exercise training sessions 7 and 18**

| **CPET and 10 minutes exercise** | | | | | |
| --- | --- | --- | --- | --- | --- |
| **CPET** | **15 minutes recovery** | **3 mins 80%VT** | **2 mins 50%∆** | **3 mins 80%VT** | **2 mins 50%∆** |

**Measurements during tests**

**List of derived variables from CPET and Spirometry**

Prior to test, Spirometry for:

- Forced Expiratory Volume in 1 Second (FEV1)
- Forced Vital Capacity (FVC)
- Inspiratory Capacity (IC)
- Calculated MVV (FEV1 * 40).

Cardio-respiratory and work variables recorded at rest and continuously throughout the test:

- Heart rate (HR)
- Blood pressure (BP)
- Oxygen saturation (SaO2)
- Tidal volume (Vt)
- Respiratory rate (RR)
- Minute ventilation (Vt*RR)
- Work rate (in Watts, from cycle ergometer)

Measures of Exercise capacity

- Lactate threshold (LT): absolute and ml/Kg – before NACRT, after NACRT, change (before NACRT –after NACRT)
- Peak Oxygen consumption (VO2 peak): absolute and ml/Kg – before NACRT, after NACRT, change (before NACRT – after NACRT)

Indicators suggestive of cardiac limitation to exercise capacity at LT:

- Oxygen pulse at LT
- Early ischaemia (before LT) on 12-lead ECG

Indicators suggestive of cardiac limitation to exercise capacity at VO2 peak:

- HR reserve at VO2 peak
- Late ischaemia (after LT) on 12-lead ECG

Indicators suggestive of respiratory limitation to exercise capacity at LT:

- Vt%/predicted IC at LT
- Indicators suggestive of respiratory limitation to exercise capacity at LT: Ventilatory reserve at VO2 peak

Ventilatory equivalent for O2 (VE/VO2) and CO2 (VE/VCO2)

- at LT
- at VO2Peak
- Calculated using the slope of the relationship throughout the CPET test

Oxygen pulse at LT and VO2Peak

AND:

VO2/WR slope

Borg scores for Breathlessness and Leg Fatigue

**List of derived variables from Exercise Intervention**

- Heart Rate
- Heart Rate Variability
- Work Load (Watts)

CT and MRI scans of the abdomen and pelvis – The first set of imaging at 9 weeks post-chemoradiotherapy is part of the clinical service. The second MRI scan at 14 weeks post-chemoradiotherapy is to assess any further down staging of the tumour, and to assess if there is any further ongoing chemoradiotherapy benefit.

Outcome Data

Post-Operative Morbidity Survey (POMS) collected at day 3, 5, 7 and 14 post-surgery

Quality of life questionnaires EQ-5D, EORCT v3 and SF-36

Hospital Resource utilization

**Health related Quality of Life Interviews**

Participants will partake in three interviews captured on audiotape. Interviews will take place before (week 1), during (week 3), and after (week 6) participation in the exercise training program. Participants will have the right to withdraw at any point from the interviews without penalty or having to give an explanation. Interview transcripts will be analyzed using thematic analysis (Strauss & Corbin, 1998), which aims to identify central themes that reflect people’s experiences.

# Interview Guide

### Interview 1

### Getting to know the participant and their experiences with colorectal cancer and exercise

1.   Tell me about yourself and your experiences with colorectal cancer?

2.   How would you describe yourself before your diagnosis?

3.   How would you describe yourself now?

4.   How did you feel when you learned of your diagnosis?

5.     Can you tell me about your experiences with chemo-radiotherapy?

6.     How are you coping with the effects of the treatment?

7.     What changes (i.e., physical/psychological) have you been experiencing?

8.     What does it mean to you to feel physically and psychologically healthy?

9.  What does it mean to you to exercise?

10. Can you tell me about your past experiences with exercise?

11. What does it mean to you to participate in the exercise training program at Aintree Hospital?

12. What thoughts will accompany you while you participate in the exercise training program?

13. How do you want to feel as you participate in the exercise training program?

14. What are your expectations regarding the exercise training program?

15. Why have you decided to participate in the program?

16. How important is it for you to participate in the program?

17. What will it mean for you to complete the program?

18. Do you have any concerns regarding your participation in the program? If yes, explain.

Interview 2

**Exploring experiences during participation in exercise program**

1.     How are you feeling at this moment?

2.     Describe your experience participating in the exercise program?

3.     What does it mean to you to participate in the program?

4.     How are you feeling as a result of your participation in the program?

5.     What thoughts are accompanying you while you participate in the program?

6.     Do you feel capable of performing the exercise program? Explain

7.     What are your expectations regarding the exercise training program?

8.     How important is it for you to participate in the exercise training program?

9.     What will it mean for you to complete the exercise training program?

10.     What does exercise mean to you?

11.     Is your participation in this program contributing to your physical and/or psychological health? If yes, how?

12.     What does it mean to you to feel physically and psychologically healthy?

13.     Do you have any concerns regarding your participation in the exercise training program? If yes, explain.

Interview 3

**Exploring experiences after participation in exercise program**

1.     How are you feeling at this moment?

2.     Describe your experiences participating in the exercise training program?

(a)     How did you feel?

(b)     What did you think?

(c)     What did you do to overcome physical challenges you may have faced?

(d)     Has this experience contributed to your physical and/or psychological health? If yes, how?

3.     Has the experience of participating in the exercise program changed you in any way? If yes. Explain.

4.     What does exercise mean to you?

5.     What have you learned about yourself so far as a result of your participation in the exercise program?

6.     Describe your thoughts and feelings relating to your upcoming surgery?

7.     Can you describe your overall thoughts and perceptions of the exercise training program?

8.     Can you tell me about your reasons for being interested or not interested in the exercise program?

9.     Did you feel capable of performing the exercise program? Explain.

10.     Do you have any concerns regarding your participation in the exercise training program? If yes, explain.

11.     Would you make any changes to the exercise program? If yes, explain.

12.     What types of exercise do you think would appeal to other colorectal cancer patients after chemo-radiotherapy?

13.     Would you once again participate in the program? Why/why not?

14.     Do you have any ideas or proposals for program improvements? If yes, explain.

Annex 1 – Blood Sampling Protocol

***Whole Blood Centrifugation***

- **Collect venous blood in 3.5 mL (lavender) vacutainers.**
- **Gently invert five times to ensure adequate mixing.**
- **Immediately place in ice for transportation to laboratory for processing.**
- **Spin at 1,000 × *g* (not rpm) for 8 minutes at room temperature; keep delay between blood draw and centrifugation short and consistent).**

**In case of hemolysis, which will cause problems with later analysis, reduce centrifugation speed to 800 x g and spin for 10 minutes. Apart from the degree of suction and needle diameter used for blood sampling, this will depend on the acceleration speed/characteristics of the centrifuge used; so, please test beforehand.**

***Plasma***

- **Immediately after centrifugation isolate plasma/supernatant by carefully pipetting off the supernatant without disturbing the buffy coat (white cell layer atop the RBC pellet).**
- **From the 1^st^ vacutainer, transfer plasma into a fresh cryovial and snap freeze in liquid nitrogen.**
- **From the 2^nd^ vacutainer, transfer plasma into 2 fresh cryovials and snap freeze in liquid nitrogen.**

***RBC Pellet***

- **Pipette off and discard the remainder of the plasma layer from each tube (containing the buffy coat) by gentle re-suspension of the liquid by forward/backward pipetting. Discard all of this.**
- **To obtain the RBC pellet, cut the tip off a normal 1mL pipette tip to provide a larger orifice through which to aspirate the pellet.**
- **Insert the pipette tip about halfway down the pellet, stir gently and start aspirating SLOWLY (to avoid contaminating your pipette).**
- **Pipette the RBCs into a fresh cryovial and repeat to maximize harvest (volumes may not be exact because it’s a little tricky to get that thick soup out of the tube, but that’s OK).**
- **Transfer remaining RBC pellet into a fresh cryovial and snap freeze in liquid nitrogen.**

**Sample Storage**

- **Keep samples frozen at least at -80ºC for no longer than 3 months.**
- **Send plasma in one go on dry ice via courier to the University of Warwick for analysis of nitrite/nitrate and nitroso content and other biomarkers.**
- **Keep RBC pellet for later analysis in a local freezer until requested.**

**SAMPLE PREPARATION FOR GENETIC MARKERS**

- **Collect venous blood in one 3.5 mL (lavender) vacutainer.**
- **Gently invert five times to ensure adequate mixing.**
- **Immediately place in ice for transportation to laboratory for processing.**
- **Transfer into cryovial and snap freeze as whole blood in liquid nitrogen.**
